# Food-Level Analysis to Identify Dietary Choices With the Highest Nutritional Quality and Lowest Greenhouse Gas Emissions and Price

**DOI:** 10.3389/fnut.2022.851826

**Published:** 2022-04-27

**Authors:** Magaly Aceves-Martins, Ruth L. Bates, Leone C. A. Craig, Neil Chalmers, Graham Horgan, Bram Boskamp, Baukje de Roos

**Affiliations:** ^1^The Rowett Institute, University of Aberdeen, Aberdeen, United Kingdom; ^2^Institute of Applied Health Sciences, University of Aberdeen, Aberdeen, United Kingdom; ^3^Biomathematics and Statistics Scotland, Aberdeen, United Kingdom; ^4^Biomathematics and Statistics Scotland, The King’s Buildings, Edinburgh, United Kingdom

**Keywords:** nutritional quality, sustainability, GHGE (greenhouse gas emissions), national diet and nutrition survey (NDNS), food prices

## Abstract

**Introduction:**

Food systems are challenged to provide healthy, sustainable and affordable foods. From a consumer perspective, identifying healthy, sustainable and affordable choices based on individual food products rather than diets could promote better shopping choices.

**Objective:**

To identify foods and drinks with the highest nutritional quality and lowest greenhouse gas emissions (GHGE) and price. We also assessed how a combination of these indicators (e.g., nutritional quality, GHGE and price) for food categories aligned with current United Kingdom dietary recommendations.

**Materials and Methods:**

We performed a secondary analysis of the National Diet and Nutrition Survey (NDNS) nutrient databank year 11 (2018/2019). Spearman correlation coefficients were used to assess the strength of relationships between nutritional quality, environmental impact and/or prices per 100 kcal. In addition, we developed an optimized nutritional quality, GHGE and price score for each food or drink item based on the overall medians for each of these indicators.

**Results:**

Median nutritional value was highest for fruit and vegetables, whilst median GHGE and price was lower for starchy carbohydrates, fats and items of which consumption should be limited. The relative proportions of foods considered the most nutritious and with a low GHGE and price in each of the food categories, on a per 100 kcal basis, were comparable to the proportions in the Eatwell Guide, except for the proportion of fruits and vegetables being smaller and the proportion of potatoes, bread, rice, pasta, and other starchy carbohydrates being larger in our analysis.

**Conclusion:**

Public health efforts should consider the impact of dietary choices not only in terms of nutritional quality but also in terms of environmental and economic impact. Our food-based analysis shows a large variation in nutritional quality, GHGE and price within and across food categories, which provides consumers with opportunities for “food swaps” that are more nutritious and have lower GHGE and price.

## Introduction

Increasingly, food systems are challenged to provide healthy, sustainable, and affordable foods for all to address the high burden of dietary-related diseases worldwide and address the environmental impact of dietary shifts (1–3). Adequate human diets need to consider nutritional balance, healthiness and address cultural acceptance, safety, access, economic fairness, and affordability. Furthermore, diets need to be protective and respectful of biodiversity and ecosystems while optimizing natural and human resources (4, 5). This requires a different approach for dietary guidelines to consider not only nutritional but also economic affordability, environmental impact, and cultural acceptability (6–8).

Dietary changes, especially those related to the amount and type of meat consumed, could reduce greenhouse gas emissions (GHGE) and land use demand by up to 50% (9). Diets high in plant-based foods are generally associated with lower GHGE and more beneficial health outcomes (10). Conversely, dietary patterns with lower GHGE show no consistent relationship with higher nutritional quality or improved health outcomes, but are correlated with elevated sugar levels and lower micronutrient intake (1). Also, reduced saturated fat and salt trends are associated with reduced GHGE in diets low in animal products (1).

Other characteristics relevant to healthy and sustainable dietary patterns, such as food prices, have been mostly overlooked (11). A previous study showed that across income quintiles, United Kingdom diets had similar GHGE, but the source of GHGE differed by types of food (e.g., meat or fruit and vegetables) (3). The dietary changes required to simultaneously improve GHGE and health outcomes were similar across income groups, including reducing animal-based products and increasing plant-based foods, with variations in specific foods depending on the income quintiles. Optimization studies have suggested that GHGE can be lowered for diets that still meet nutritional requirements (12, 13). Nevertheless, difficulties in changing eating habits to meet dietary improvement recommendations or national dietary guidelines have been reported (14).

Most studies thus far have considered the optimization of whole diets in terms of nutritional quality and environmental sustainability. Nevertheless, from a consumer’s perspective, identifying healthy, sustainable, and adequate food prices choices based on individual food products rather than whole diets could promote better shopping choices (15, 16). Previous evidence has suggested that a strategy based on simple substitutions within food subgroups or “food swaps” is effective in rapidly improving the nutritional adequacy of the diet (14, 17, 18). Recent research from the United States showcase that “food swaps” can substantially reduce environmental markers (e.g., carbon and water scarcity footprints) without compromising dietary quality (16). However, none of these studies has considered the environmental impact and food prices simultaneously. Indeed, multidimensional data on individual food and drink products is needed to optimize healthy, green, and affordable foods that could define optimal food swaps (4). This study aimed to identify food and drink items with the highest nutritional quality, the lowest GHGE and price, in the United Kingdom. We also assessed how the combination of these dimensions (nutritional quality, GHGE and price) for foods and food categories align with current United Kingdom dietary recommendations.

## Materials and Methods

### Data

We used compositional data from the nearly 6,000 commonly consumed foods and drinks, and prepared dishes, used for the National Diet and Nutrition Survey (NDNS nutrient databank) year 11 (2018/19) (19–21). Data of toddler food, infant formula, nutrition powders and supplements were removed to focus on daily food and drink items consumed by a general adult population (>18 years old).

### Food Categories

Similar to the United Kingdom food-based dietary guidelines for healthy eating (The Eatwell Guide) (22, 23), individual food and drinks from the NDNS nutrient databank were aggregated (based on their main components) into six food categories (i.e., fruit and vegetables; potatoes, bread, rice, pasta, and other starchy carbohydrates; beans, pulses, fish, eggs, meat, and other proteins; dairy and alternatives; and oils, spreads and fats; items that should be limited or eaten in small amounts). In addition, to identify foods/drinks high in fat, salt and/or sugars, the cut-off points for fat, salt, and sugar from the current United Kingdom Guide to creating a front of pack (FoP) nutrition label for pre-packed products were used (24). Cut-off points are presented in Table 1. Although the cut-off points of the FoP guidelines differ slightly from those in the Eatwell Guide, the use of FoP cut-offs was selected as it has specific limits for both food and drinks, and these are consistent with the Eatwell Guidelines (25).

**TABLE 1:** Cut-off points for fat, salt, and sugar from the current United Kingdom Guide to creating a front of pack (FoP) label.

Component	Foods	Drinks
Total Sugar	≥ 22.5 g/100 g	≥11.25 g/100 g
Total Fat	≥ 17.5 g/100 g of total fat	≥8.75 g/100 g
Total Saturated fat	≥ 5.0 g/100 g	≥2.5 g/100 g
Total Salt	≥ 1.5 g/100 g	≥0.75 g/100 g

*g = grams.*

### Indicators of Nutritional Quality, Environmental Impact, and Price

#### Nutritional Quality

The Nutrient-Rich Food Index 8.3 (NRF8.3) was calculated for all the foods and beverages in the NDNS nutrient databank for each food/drink item (26–28). This index can be estimated by 100 kcal, 100 g, or serving size. For this study, we estimated the NRF8.3 per 100 kcal of product. NRF index scores are dietary quality indices based on the nutrient density of a food item, accounting for beneficial nutrients, nutrients to limit, or a combination of both. This index is estimated as NRn - LIM = NRFn.3, where n represents the number of nutrients to encourage and LIM represents three nutrients that should be limited (29). The number of qualifying nutrients to encourage has varied across different studies, from 6 (NRF 6.3) to 15 (NRF 15.3) (29). In the current work, we adapted the original NRF9.3 model to a NRF8.3 model since there is no United Kingdom reference value for vitamin E, and therefore this could not be included in the model. Hence the qualifying nutrients in our study were: protein, fiber, vitamins a and c, calcium, iron, magnesium and potassium, while the disqualifying were saturated fat, total sugars and sodium. The higher the scores, the better nutritional quality (26–28).

#### Environmental Impact

GHGE values for individual foods and dishes, expressed as gCO_2_-equivalents (CO_2_e), were obtained from open-access sources published between 2008 and 2016 and added to the NDNS nutrient databank (30, 31). In addition, GHGE values from studies using complete cradle-to-grave life cycle analysis (LCA) (30), obtained following the international PAS 2050 standard (32), were selected where possible. In this report (30), to express a product’s carbon footprint as a single number, the emissions of six greenhouse gases were converted into an equivalent amount of carbon dioxide (CO_2_ equivalent or CO_2_e), based on the relative global warming impact of each gas, and the final carbon footprint is expressed as the weight of carbon dioxide. The climate metric used to aggregate the GHGE measurements into CO_2_e were those reported by Department for Environment Food and Rural Affairs, United Kingdom (33). We identified CO_2_e for 153 food and drink items in the open-access databases. Where a GHGE value for a specific item was not available, which was the case for most of the food and drinks in our database, reasonable substitute data were discussed and agreed upon by a team of 3 nutrition scientists, based on the food type, food group and compositional similarity of the products where data was available (e.g., 320 CO_2_e was identified for spaghetti, hence for most of the pasta products CO_2_e of 320 was used).

#### Prices

Prices (in GBP) of items in the NDNS nutrient databank were retrieved up to October 2021. The Shelf Scraper search engine was used to search for individual food and drink items prices (34), or prices were searched manually on supermarket websites if not available from the search engine. The Shelf Scraper website considers Tesco, ASDA, Sainsbury’s, and Morrison’s prices (the largest and most frequently used supermarkets in the United Kingdom). This website’s prices are updated daily, not aggregated across retailers, and sorted by unit to compare retailers side-by-side (34). A standard weight was estimated from nutritional guidelines for items where the price was provided per portion rather than weight. Also, prices were adjusted per edible portion of the food products. The retail prices were used and were not adjusted for inflation, and the lowest price between supermarkets was used. The price of cooking was not considered within the price recorded. All the prices were obtained per 100 g and then estimated per 100 kcal of product. The prices of 4432 products were retrieved (90% of the items included in the analysis).

### Analysis

Nutritional quality (NRF8.3 index), GHGE (in g CO_2_e) and price (in GBP) of all food and drinks available in our expanded NDNS nutrient databank were calculated per 100 kcal of food/drink item. Shapiro–Wilk tests were performed for each indicator across the food and drinks categories to evaluate the normality of data and suggested significant non-normality among categories for all three indicators (i.e., nutritional quality, GHGE and price) (Supplementary Appendix). Hence, a non-parametric test (Spearman’s correlation) was selected to assess the strength of relationships between nutritional quality, environmental impact and/or prices by food category and subcategory. The correlations were defined using the following values: 0.00-0.19 “very weak”; 0.20-0.39 “weak”; 0.40-0.59 “moderate”; 0.60-0.79 “strong”; 0.80-1.0 “very strong” (35). Raw p-values were analyzed using the Bonferroni correction for each analysis food group and subgroups to control the family-wise error rate. Hence, the statistical significance varied depending on food groups and subgroups analysis.

Following methods previously described (6, 15), a combined score based on nutritional quality, GHGE and price for each food or drink item was developed, based on the overall medians for each indicator. The scoring system ranged from 0 to 3, with each food and/or drink scoring 1 point if the NRF8.3 index score was above the median, 1 point if its GHGE were under the median and 1 point if its price was under the median. Those items with the highest score (i.e., 3) represented the food items most nutritious and with the lowest GHGE and price. This analysis was performed on a per 100 kcal basis. The proportion (%) of items with the highest score on a 100-kcal basis were tabulated according to their food category. Such a distribution was also graphically presented in a pie chart, using the Eatwell Guide’s color scheme for comparison purposes.

Analysis was done in R software using the libraries “*ggplot2*”, “*ggthemes*”, “*tidiverse*” (for data visualization and graphs), *“dply*r” (for testing normality), “*psych*” and “*pastecs*” (for descriptive statistics).

## Results

Of the 5,927 items included in the NDNS nutrient databank, 819 were irrelevant for our analysis (e.g., toddler food or baby formulas) and were removed. An additional 198 items (e.g., artificial sweeteners, cooking spices, and dried herbs) were removed as they were not linked to any food and drink categories. Therefore, a total of 4,910 food and drinks from the NDNS nutrient databank were included in this analysis; 16% of items were categorized as fruit or vegetables; 28% were categorized as potatoes, bread, rice, pasta and other starchy carbohydrates; 34% were categorized as beans, pulses, fish, eggs, meat and other proteins; 8% were categorized as dairy and alternatives; 2% were categorized as oils and spreads; 6% were categorized as drinks, and 6% items were categorized as those that should be avoided or eaten in fewer amounts (Table 2).

**TABLE 2:** Distribution of food and drinks per category and subcategory in the NDNS nutrient databank.

Category	Total number of items	Subcategory	Number of items (% within a category)	Number of items with a high content of sugar, fat and/or salt* (% within a subcategory)
Fruit and vegetables	800	Fruits	183 (23%)	44 (24%)
		Vegetables	262 (33%)	28 (11%)
		Juices and fruit canned in juice	199 (25%)	26 (13%)
		Prepared dishes/takeaway based on fruit or vegetables	156 (19%)	27 (17%)
Potatoes, bread, rice, pasta, and other starchy carbohydrates	1378	Cereals	509 (37%)	161 (32%)
		Potatoes	163 (11%)	21 (13%)
		Prepared dishes/takeaway based on cereals	706 (52%)	481 (68%)
Beans, pulses, fish, eggs, meat, and other proteins	1689	Beans and pulses	141 (8%)	9 (6%)
		Seeds and Nuts	51 (3%)	47 (92%)
		Oily fish	103 (6%)	49 (48%)
		White fish or shellfish	254 (15%)	59 (23%)
		Meats	500 (30%)	280 (56%)
		Eggs	53 (3%)	21 (40%)
		Prepared dishes/takeaway based on animal proteins (not canned)	587 (34%)	164 (28%)
Dairy and alternatives	380	Milk	46 (12%)	0 (0%)
		Alternative Milks (non-animal)	29 (8%)	2 (7%)
		Cheese	86 (22%)	79 (91%)
		Yogurt	59 (16%)	6 (10%)
		Other dairy products and desserts	160 (42%)	91 (59%)
Oils, spreads, and fats	84	Vegetable oils and vegetable-based spreads	67 (78%)	67 (100%)
		Animal fats	17 (22%)	17 (100%)
Drinks	301	Soft drinks	172 (47%)	50 (29%)
		Coffee and Tea	37 (18%)	5 (14%)
		Alcohol	92 (35%)	18 (20%)
Items which should be eaten less often and in small amounts	278	All products (e.g., sugar confectionery, sweet spreads fillings, icing, savory sauces, pickles gravies, crisps, and savory snacks)	278 (100%)	278 (100%)

A significant strong positive correlation (ρ = 0.66) between food price and NRF8.3 for fruits indicated that better nutritional quality comes at a higher price. There was also a significant strong to very strong positive correlation between price and GHGE for the following food sub-groups: beans and pulses, oily fish, white fish coated, other white fish, shellfish, and fish dishes, soft drinks, coffee, and tea per 100 kcal of product, showing that lower prices were associated with lower GHGE for these products. In addition, there was a significant strong positive correlation between NRF8.3 and GHGE for alcohol per 100 kcal of product, indicating that for these products, higher nutritional quality is associated with higher GHGE (Table 3).

**TABLE 3:** Correlation between NRF8.3, GHGE, and price indicators.

Category	NRF8.3 & Price	NRF8.3 & GHGE	Price & GHGE	Subcategory	NRF8.3 & Price	NRF8.3 & GHGE	Price & GHGE
All items	0.30*	0.38*	0.55*	NA	NA	NA	NA
Fruit and vegetables	0.49*	0.47*	0.55*	Fruits	0.66*	0.58*	0.53*
				Vegetables	0.48*	0.53*	0.54*
				Juices & fruit canned in juice	0.31*	0.18	0.59*
				Prepared dishes/takeaway based on fruit or vegetables	0.34*	0.40*	0.50*
Potatoes, bread, rice, pasta, and other starchy carbohydrate	−0.01	0.45*	0.24*	Cereals	0.22*	0.12	0.33*
				Potatoes	0.16	0.64*	0.30*
				Prepared dishes/takeaway based on cereals	0.27*	0.53*	0.35*
Beans, pulses, fish, eggs, meat, and other proteins	0.21*	0.27*	0.45*	Beans and pulses	0.09	0.24	0.65*
				Seeds and Nuts	0.15	0.11	0.25
				Oily fish	0.11	0.41*	0.63*
				Whitefish coated or fried and other white fish, shellfish, and fish dishes	0.42*	0.21*	0.61*
				Meats	0.29*	0.50*	0.47*
				Eggs	0.26	0.11	0.82*
				Prepared dishes/takeaway based on animal proteins (not canned)	0.45*	0.44*	0.37*
Dairy and alternatives	0.27*	0.11	0.24*	Milk	0.35	−0.23	0.03
				Milk Alternatives (non-animal)	0.43	0.16	0.43
				Cheese	0.15	0.15	0.42*
				Yogurt	0.37	0.29	0.22
				Other dairy products and desserts	0.33	0.17	0.44
Oils, spreads, and fats	0.39*	0.50*	0.59*	Vegetable oils and vegetable-based spreads	0.26	0.04	0.27
				Animal fat	0.57	0.10	0.30
Drinks	0.22*	0.48*	0.71*	Soft drinks	0.19	0.45*	0.66*
				Coffee & Tea	−0.12	−0.01	0.68*
				Alcohol	0.21	0.78*	0.37*
Items that consumption should be limited	0.25*	0.45*	0.29*	All products (e.g., Sugar confectionery, sweet spreads fillings, icing, savory sauces pickles gravies, crisps, and savory snacks)	0.25*	0.45*	0.26*

NRF8.3, Nutrient-Rich Food Index 8.3; GHGE, greenhouse gas emissions. Price calculated in British Pounds (GBP). Raw p values were analyzed using Bonferroni correction, and * show those values that were considered statistically significant; the strength of the correlation was defined using the following values: 0.00-0.19 “very weak”; 0.20-0.39 “weak” 0.40-0.59 “moderate”; 0.60-0.79 “strong”; 0.80-1.0 “very strong”.

**FIGURE 1:**
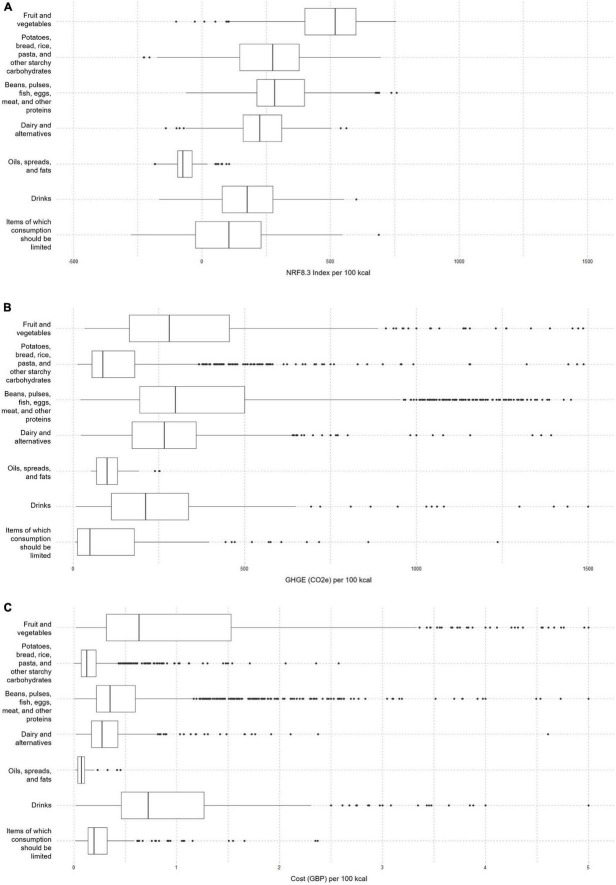
**(A)** The overall NRF 8.3 median across all the food and beverage items included in the analysis was 287/100 kcal. The medians per food categories were as follows: fruit and vegetables 507/100 kcal; potatoes, bread, rice, pasta, and other starchy carbohydrates 275/100 kcal; beans, pulses, fish, eggs, meat, and other proteins 283/100 kcal; dairy and alternatives and 225/100 kcal; oils and spreads and animal fats -72/100 kcal; Drinks 184/100 kcal; and foods high in fat, salt, or sugar 124/100 kcal. **(B)** The overall GHGE median across all the food and beverage items included in the analysis was 211/100 kcal. The medians per food categories were as follows: fruit and vegetables 281/100 kcal; potatoes, bread, rice, pasta, and other starchy carbohydrates 87/100 kcal; beans, pulses, fish, eggs, meat, and other proteins 304/100 kcal; dairy and alternatives 262/100 kcal; oils and spreads and animal fats 105/100 kcal; Drinks 217/100 kcal; and foods high in fat, salt, or sugar 65/100 kcal. **(C)** The overall median across all the food and beverage items included in the analysis was £0.30/100 kcal. The medians per food categories were as follows: fruit and vegetables £0.64/100 kcal; potatoes, bread, rice, pasta, and other starchy carbohydrates £0.13/100 kcal; beans, pulses, fish, eggs, meat, and other proteins £0.36/100 kcal; dairy and alternatives £0.27/100 kcal; oils and spreads and animal fats £0.08/100 kcal; drinks £0.24/100 g and £0.83/100 kcal; and foods high in fat, salt, or sugar £0.20/100 kcal.

Median values for nutritional quality were generally highest in the categories of fruits and vegetables; potatoes, bread, rice, pasta, and other starchy carbohydrates; and beans, pulses, fish, eggs, meat, and other proteins per 100 kcal of product. Conversely, median values for GHGE and price were generally lowest for potatoes, bread, rice, pasta and other starchy carbohydrates, oils spreads and fats, and items for which consumption should be limited). (See Table 3 for results per food groups and sub-groups). In addition, for some food categories, the variation around the medians for NRF8.3 index, GHGE and price was relatively large, showing that within food categories, some individual foods and/or drinks could score much better or worse than others, highlighting the potential for food swaps (Figure 1).

**FIGURE 2:**
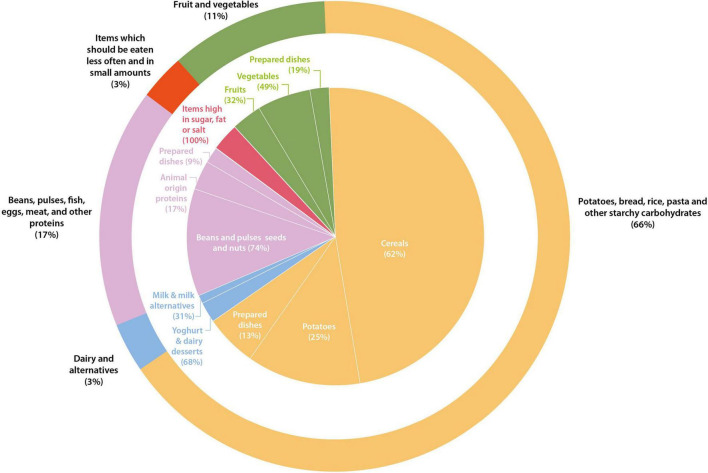
Food groups designated following the Eatwell Guide groups. Each food scored 1 point if its GHGE was under the median, 1 point if its price was under the median, and 1 point if its nutritional score was above the median for the relevant food group. This graph shows those items with the highest score (scoring 3) and showcase the most environmentally sustainable, nutritious, and lower price products per every 100 kcal. The outer graphs show the overall percentages, and the inner graph subdivides the information per food sub-groups.

The proportion (%) of foods and drinks within each of the principal and sub-food categories scoring the maximum possible (i.e., 3) for the three indicators (scoring 1 point each for being above the median for the NRF8.3 index, and scoring 1 point each for being below the medium for GHGE and price), on a 100 kcal basis, was highest for the categories potatoes, bread, rice, pasta and other starchy carbohydrates, and lowest for the category of dairy and alternatives (Figure 2). No product from the oils, spreads and fats or drinks scored the maximum possible score. Some examples of the most nutritious, environmentally sustainable, and lower price food and drink items are presented in Table 4. Overall, the vast majority (66.2%) of products scoring 3 points were part of the potatoes, bread, rice, pasta, and other starchy carbohydrates food group. Most of the products from the beans, pulses, fish, eggs, meat and other proteins (96 products representing 16.6% of the overall number of products scoring 3 points) were from a non-animal origin (i.e., beans and pulses or seeds and nuts). Only 10.9% of the products scoring 3 points were part of the fruits and vegetables food group, and over half of these products were considered to have a high content of salt, fat, saturated fat, or sugar.

**TABLE 4:** Most nutritious, environmentally sustainable, and lower price products.

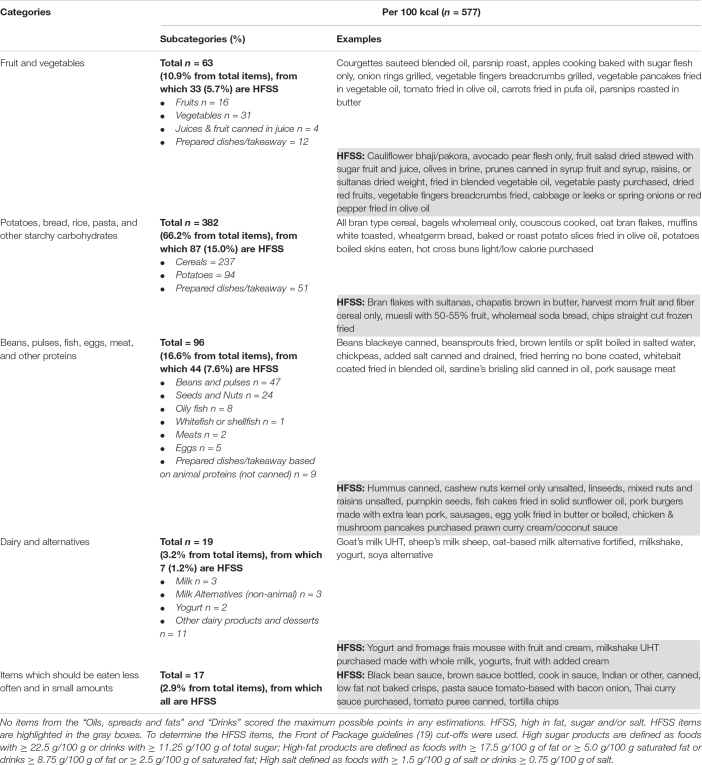

## Discussion

Our food-based analysis showed that the largest proportion of the food items that were most nutritious and with the lowest GHGE and price was found in potatoes, bread, rice, pasta, and other starchy carbohydrates, whilst the smallest proportion was found in the category of dairy and alternatives. Generally, the relative proportions of most nutritious, environmentally sustainable, and lowest-priced foods in each of the seven food categories, analyzed through a food-based approach on a per 100 kcal basis, were relatively similar to the Eatwell Guide’s proportions, which takes a diet-based approach. However, there were two crucial differences - whilst the category of fruit and vegetables is a main category in the Eatwell Guide, this category was much smaller in our food-based analysis. Furthermore, within categories and subcategories, a large variation in nutritional quality, GHGE and price suggested significant scope for optimizing “food swaps” to improve all three indicators.

In the United Kingdom, government dietary recommendations come in dietary reference values and food-based guidelines represented in the Eatwell Guide, which has evolved over the years to visually represent the types and proportions of foods needed for a healthy balanced diet (25). The current recommendations were updated in 2016 to ensure consistent dietary recommendations with critical public health messages (e.g., obesity prevention, limiting free sugar consumption, and increasing fiber consumption). Although health and nutritional quality were the primary focus in the last revision of the guidelines, environmental sustainability was also accounted for to some extent (8, 25, 36). More recently, the Carbon Trust was commissioned to conduct a sustainability assessment of the Eatwell Guide, which indicated that eating a diet in line with the Eatwell Guide has a substantially lower environmental impact than the current United Kingdom diet (37, 38). Also, a recent study using empirical data concluded that greater adherence to the Eatwell Guide recommendations is associated with health and environmental benefits (39).

In our study, around 12% of the items from the NDNS nutrient databank obtained the highest score of 3 and were considered the most nutritious, and with a lower GHGE and price (Figure 2). Our results are in line with the results presented by Masset et al. (15), providing helpful insights into the relationship between the environmental impact, nutritional quality, and price of individual foods. Interestingly, our food-based analysis, including price (in addition to GHGE and NRF8.3) as an additional variable, shows that examples of foods that are identified as most nutritious, and with a lower GHE and price, include a relatively high number of products that are high in fat, sugar, and salt/or salt on a per 100 kcal basis (Table 4). According to the current dietary guidelines, such products should be eaten less often and in small amounts. Some studies have indicated that foods high in fat, sugar, and/or salt could be cheaper and have a lower environmental impact (40) and might be consumed in higher amounts. Nevertheless, one of the main limitations of a food-level basis is the lack of consideration of the consumption patterns in the population, and food-based analysis may to be complemented with diet-based modeling approaches to appreciate the complexity of the relationship between nutritional quality, GHGE and price. Thus, we identified a relatively high number of products with high fat, sugar, and salt/or salt content, but these foods may only contribute to an overall healthy and sustainable diet if consumed in small quantities.

An advantage of our analysis is that we introduced the food prices along with the nutritional quality and the estimated GHGE up to the supermarket shelves. Our results showed a strong positive correlation between nutritional quality and the price of fruits. It has been estimated that a 2,000 kcal-diet for an adult costs on average £5.54 per day in the United Kingdom (41). However, those diets meeting recommendations for fruit and vegetables, or oily fish, are 16% to 17% more expensive (41). Also, it is estimated that 27% of the United Kingdom households would need to spend more than a quarter of their disposable income on a diet that conforms with the Eatwell Guide (42). Accounting for food prices is crucial since consumers persistently cited this as the most important determinant of food choice in the United Kingdom (43, 44). The price has also been emphasized as a population-level barrier, limiting the adoption of dietary recommendations in the United Kingdom (41, 45), especially for those with lower socioeconomic status (45, 46). Indeed, the price is a pivotal contributor to socioeconomic inequalities in food and drink choices’ healthiness (46, 47).

A notably low amount of animal-based protein products was among foods that were most nutritious and with the lowest GHGE and price (Table 4). This reinforces the message that reduced consumption of animal-based products would represent a substantial switch to make diets more sustainable (48). Moreover, our data show that a higher nutritional quality in meat products is positively correlated with GHGE and price (Table 3). The need to produce environmentally sustainable, economically affordable and nutritious foods presents a current and future challenge to food systems (49). However, because of the complexity of what a “sustainable diet” comprises, it is challenging to consider all the relevant characteristics simultaneously.

Some limitations of the present work include the high number of imputations made in GHGE data. Also, whilst the GHGE data available for the food and drink items in our database are typically linked across each product across the entire life cycle (30, 50), it does not include cooking methods (51), mean blue water foot print (39) or international trade (52), which would be relevant for a range of items included in our analysis. Indeed, by using alternative climate metrics than GHGE alone we may have come to different conclusions. In addition, we did not consider consumption frequency, which would be relevant when estimating food preferences and cultural acceptance (15).

In this study, data was analyzed on a 100-kcal basis, which is the unit of the NRF8.3 index. Although counter-intuitive, a high NRF8.3 index can also be associated with foods high in fat, salt, and sugar, depending on how they are expressed (per 100 kcal or 100 g) (40, 53). Drewnoski et al. (53) found that nutritional profiles based on per 100 g of product are more consistent with food labeling frameworks but penalize energy-dense foods consumed in small quantities (e.g., nuts or seeds or a) while giving excessively favorable scores to foods containing added sugar (or fat or salt, as our study shows) that are mostly consumed in volumes over 100 g. By using energy basis unit (kcal), some GHGE and price results might favor high-energy food items. Also, by using NRF8.3, we might overlook the role of essential nutrients in the nutritional quality of foods. Nevertheless, several measurements, such as food products’ weight or energy density, can be included to compare results in future research.

Strengths of the present work include analyzing nearly 4,900 food and drinks items, including ready meals and purchased foods, available in the United Kingdom. To our knowledge, this is the first food-level study in the United Kingdom to include NDNS nutrient databank data on nutritional quality, GHGE and price simultaneously, offering a food-based model approach that allowed the review of nutritional quality, sustainability, and price of the suggested proportions of foods and drinks in the Eatwell Guide. This food-based modeling approach allowed the identification of combinations optimized in terms of nutritional quality, GHGE and price for individual food or drink items, which could ultimately result in modeling food swaps to make individual diets healthier and more sustainable whilst considering food prices.

Identifying individual healthy and sustainable foods at a lower price rather than diets could promote better shopping choices, as consumers could make informed choices about practical food swaps.

In conclusion, we showed for the first time that the relative proportion of foods and drinks in the United Kingdom that are nutritious, and with a low GHGE and price are mostly in line with current recommendations, apart from the proportion of fruit and vegetables being smaller in our analysis because of its generally higher values and variability for GHGE and price, and the proportion of potatoes, bread, rice, pasta, and other starchy carbohydrates being larger in our analysis, becoming more prominent due to its generally lower prices and high nutritional value. Our work highlights the importance of simultaneously considering all three indicators (e.g., nutritional quality, GHGE, and price) when making food choices. All these three indicators can be considered critical factors for dietary behavior change. In future research, other indicators need to be included (e.g., acceptability and culturally acceptable choices). Simultaneous modeling of these indicators also offers new opportunities to identify “food swaps”, permitting individuals to make their diets healthier and greener whilst not necessarily compromising on price.

## Data Availability Statement

The datasets presented in this article are not readily available because described in the manuscript and R code will be available upon request pending application and approval from the authors and the institute. Requests to access the datasets should be directed to BR, b.deroos@abdn.ac.uk.

## Author Contributions

MA-M and BR formulated the research question and designed the study. All authors were involved in collecting the relevant data used in the analysis of this study. In addition, all authors contributed to writing the article.

## Conflict of Interest

The authors declare that the research was conducted in the absence of any commercial or financial relationships that could be construed as a potential conflict of interest.

## Publisher’s Note

All claims expressed in this article are solely those of the authors and do not necessarily represent those of their affiliated organizations, or those of the publisher, the editors and the reviewers. Any product that may be evaluated in this article, or claim that may be made by its manufacturer, is not guaranteed or endorsed by the publisher.

## References

[1] Payne CL Scarborough P Cobiac L. Do low-carbon-emission diets lead to higher nutritional quality and positive health outcomes? A systematic review of the literature. *Public Health Nutr.* (2016) 19:2654–61. 10.1017/s1368980016000495 26975578 PMC10270842

[2] Perignon M Vieux F Soler LG Masset G Darmon N. Improving diet sustainability through evolution of food choices: review of epidemiological studies on the environmental impact of diets. *Nutr Rev.* (2017) 75:2–17. 10.1093/nutrit/nuw043 27974596 PMC5155614

[3] Reynolds CJ Horgan GW Whybrow S Macdiarmid JI. Healthy and sustainable diets that meet greenhouse gas emission reduction targets and are affordable for different income groups in the UK. *Public Health Nutr.* (2019) 22:1503–17. 10.1017/s1368980018003774 30782231 PMC10260812

[4] Burlingame B Dernini S Nutrition and Consumer Protection Division. *Sustainable Diets and Biodiversity. Directions and Solutions for Policym Research and Action.* Rome: FAO (2010).

[5] Tuomisto HL. The complexity of sustainable diets. *Nat Ecol Evol.* (2019) 3:720–1. 10.1038/s41559-019-0875-5 30988495

[6] Masset G Vieux F Verger EO Soler LG Touazi D Darmon N. Reducing energy intake and energy density for a sustainable diet: a study based on self-selected diets in French adults. *Am J Clin Nutr.* (2014) 99:1460–9. 10.3945/ajcn.113.077958 24695893

[7] Parlesak A Tetens I Dejgård Jensen J Smed S Gabrijelèiè Blenkuš M Rayner M Use of linear programming to develop cost-minimized nutritionally adequate health promoting food baskets. *PLoS One.* (2016) 11:e0163411. 10.1371/journal.pone.0163411 27760131 PMC5070943

[8] Scarborough P Kaur A Cobiac L Owens P Parlesak A Sweeney K Eatwell guide: modelling the dietary and cost implications of incorporating new sugar and fibre guidelines. *BMJ Open.* (2016) 6:e013182. 10.1136/bmjopen-2016-013182 28003292 PMC5223664

[9] Hallström E Carlsson-kanyama A Börjesson P. Environmental impact of dietary change: a systematic review. *J Clean Prod.* (2015) 91:1–11. 10.1016/j.jclepro.2014.12.008

[10] Joyce A Hallett J Hannelly T Carey G. The impact of nutritional choices on global warming and policy implications: examining the link between dietary choices and greenhouse gas emissions. *Energy Emiss Control Technol.* (2014) 2:33–43. 10.2147/eect.s58518

[11] Springmann M Clark MA Rayner M Scarborough P Webb P. The global and regional costs of healthy and sustainable dietary patterns: a modelling study. *Lancet Planet Health.* (2021) 5:e797–807. 10.1016/S2542-5196(21)00251-5 34715058 PMC8581186

[12] Macdiarmid JI Kyle J Horgan GW Loe J Fyfe C Johnstone A Sustainable diets for the future: can we contribute to reducing greenhouse gas emissions by eating a healthy diet? *Am J Clin Nutr.* (2012) 96:632–9. 10.3945/ajcn.112.038729 22854399

[13] Green R Milner J Dangour AD Haines A Chalabi Z Markandya A The potential to reduce greenhouse gas emissions in the UK through healthy and realistic dietary change. *Clim Change.* (2015) 129:253–65. 10.1007/s10584-015-1329-y

[14] Verger EO Holmes BA Huneau JF Mariotti F. Simple changes within dietary subgroups can rapidly improve the nutrient adequacy of the diet of french adults. *J Nutr.* (2014) 144:929–36. 10.3945/jn.113.188284 24699804

[15] Masset G Soler LG Vieux F Darmon N. Identifying sustainable foods: the relationship between environmental impact, nutritional quality, and prices of foods representative of the French diet. *J Acad Nutr Diet.* (2014) 114:862–9. 10.1016/j.jand.2014.02.002 24703928

[16] Rose D Willits-Smith AM Heller MC. Single-item substitutions can substantially reduce the carbon and water scarcity footprints of US diets. *Am J Clin Nutr.* (2022) 115:378–87. 10.1093/ajcn/nqab338 35024805 PMC8827079

[17] Wrieden WL Levy LB. ‘Change4Life smart swaps’: quasi-experimental evaluation of a natural experiment. *Public Health Nutr.* (2016) 19:2388–92. 10.1017/S1368980016000513 27002189 PMC4981896

[18] Strid A Hallström E Sonesson U Sjons J Winkvist A Bianchi M. Sustainability indicators for foods benefiting climate and health. *Sustainability.* (2021) 13:3621. 10.3390/su13073621

[19] PHE. *National Diet and Nutrition Survey.* London: Public Health England (2016).

[20] PHE. *McCance and Widdowson’s The Composition of Foods Integrated Dataset 2021.* London: Public Health England (2021).

[21] University of Cambridge, MRC Epidemiology Unit, NatCen Social Research. *National Diet and Nutrition Survey Years 1-11, 2008-2019 (Dataset)*. 19 ed. UK Data Service (2021). 10.5255/UKDA-SN-6533-19

[22] Buttriss JL. The eatwell guide refreshed. *Nutr Bull.* (2016) 41:135–41. 10.1111/nbu.12211

[23] PHE. *The Eatwell Guide.* London: Public Health England (2018).

[24] FSA Department of Health. *Guide to Creating a Front of Pack (FoP) Nutrition Label for Pre-Packed Products Sold Through Retail Outlets.* London: FSA Department of Health (2016).

[25] PHE. *From Plate to Guide: What, Why and How for the Eatwell Model.* London: Public Health England (2016).

[26] Fulgoni VL Keast DR Drewnowski A. Development and validation of the nutrient-rich foods index: a tool to measure nutritional quality of foods. *J. Nutr.* (2009) 139:1549–54. 10.3945/jn.108.101360 19549759

[27] Drewnowski A. The nutrient rich foods index helps to identify healthy, affordable foods. *Am J Clin Nutr.* (2010) 91:1095S–101S. 10.3945/ajcn.2010.28450d 20181811

[28] Drewnowski A Fulgoni VL. Nutrient density: principles and evaluation tools. *Am J Clin Nutr.* (2014) 99:1223S–8S. 10.3945/ajcn.113.073395 24646818

[29] Drewnowski A Richonnet C. Dairy and fruit listed as main ingredients improve NRF8.3 Nutrient density scores of children’s snacks. *Front Nutr.* (2020) 7:15. 10.3389/fnut.2020.00015 32211416 PMC7075804

[30] TESCO. *Product Carbon Footprint Summary.* Hackney: TESCO (2012).

[31] Bates R Chambers N Craig L. Greenhouse gas emissions of UK diets. *Proc Nutr Soc.* (2019) 78:E65. 10.1017/S0029665119000910

[32] BSI. *Specification for the Assessment of the Life Cycle Greenhouse Gas Emissions of Goods and Services.* London: BSI British Standards (2011).

[33] FRA Department for Environment. *2012 Greenhouse Gas Conversion Factors for Company Reporting.* London: FRA Department for Environment (2013).

[34] Cracknell J. *ShelfScraper [Online]*. (2021). Available online at: https://shelfscraper.herokuapp.com/ (accessed October 04, 2021).

[35] Ramalingam TA Kumar SN. *Essentials of Research Methodology for all Physiotherapy and Allied Health Sciences Students.* Daryaganj: Jaypee Brothers Medical Publishers (2018).

[36] Macdiarmid JI Kyle J Horgan GW Loe JE Fyfe C Johnstone A *Livewell: a Balance of Healthy and Sustainable Food Choices.* London: World Wildlife Fund (2011).

[37] Cobiac LJ Scarborough P Kaur A Rayner M. The eatwell guide: modelling the health implications of incorporating new sugar and fibre guidelines. *PLoS One.* (2016) 11:e0167859. 10.1371/journal.pone.0167859 27997546 PMC5173361

[38] The Carbon Trust [TCT]. *The Eatwell Guide: a More Sustainable Diet Methodology and Results Summary.* London: The Carbon Trust (2016).

[39] Scheelbeek P Green R Papier K Knuppel A Alae-Carew C Balkwill A Health impacts and environmental footprints of diets that meet the eatwell guide recommendations: analyses of multiple UK studies. *BMJ Open.* (2020) 10:e037554. 10.1136/bmjopen-2020-037554 32847945 PMC7451532

[40] Poti JM Braga B Qin B. Ultra-processed food intake and obesity: what really matters for health-processing or nutrient content? *Curr Obes Rep.* (2017) 6:420–31. 10.1007/s13679-017-0285-4 29071481 PMC5787353

[41] Jones NR Tong TY Monsivais P. Meeting UK dietary recommendations is associated with higher estimated consumer food costs: an analysis using the national diet and nutrition survey and consumer expenditure data, 2008–2012. *Public Health Nutr.* (2018) 21:948–56. 10.1017/s1368980017003275 29198220 PMC5848749

[42] Scott C Sutherland J Taylor A. *Affordability of the UK’s Eatwell Guide.* London: The Food Foundation (2018).

[43] Food Statistics team. *Food Statistics Pocketbook 2016.* London: Department for Environment, Food and Rural Affairs (2016).

[44] Statistics N. *Food Statistics Pocketbook.* London: F.R.A. Department for Environment (2020).

[45] Morris MA Hulme C Clarke GP Edwards KL Cade JE. What is the cost of a healthy diet? Using diet data from the UK Women's cohort study. *J Epidemiol Commun Health.* (2014) 68:1043. 10.1136/jech-2014-204039 25053614

[46] Pechey R Monsivais P. Socioeconomic inequalities in the healthiness of food choices: exploring the contributions of food expenditures. *Prev Med.* (2016) 88:203–9. 10.1016/j.ypmed.2016.04.012 27095324 PMC4910945

[47] Pechey R Monsivais P Ng YL Marteau TM. Why don’t poor men eat fruit? Socioeconomic differences in motivations for fruit consumption. *Appetite.* (2015) 84:271–9. 10.1016/j.appet.2014.10.022 25451584 PMC4262578

[48] Willett W Rockström J Loken B Springmann M Lang T Vermeulen S Food in the anthropocene: the EAT–lancet commission on healthy diets from sustainable food systems. *Lancet.* (2019) 393:447–92. 10.1016/s0140-6736(18)31788-4 30660336

[49] Drewnowski A. Analysing the affordability of the EAT–lancet diet. *Lancet Glob Health.* (2020) 8:e6–7. 10.1016/s2214-109x(19)30502-9 31839142

[50] Jones AD Hoey L Blesh J Miller L Green A Shapiro LF. A systematic review of the measurement of sustainable diets. *Adv Nutr Int Rev J.* (2016) 7:641–64. 10.3945/an.115.011015 27422501 PMC4942861

[51] Frankowska A Rivera XS Bridle S Kluczkovski AMRG Tereza da Silva J Martins CA Impacts of home cooking methods and appliances on the GHG emissions of food. *Nat Food.* (2020) 1:787–91. 10.1038/s43016-020-00200-w 37128063

[52] Sandström V Valin H Krisztin T Havlík P Herrero M Kastner T. The role of trade in the greenhouse gas footprints of EU diets. *Glob Food Sec.* (2018) 19:48–55. 10.1016/j.gfs.2018.08.007

[53] Drewnowski A Maillot M Darmon N. Should nutrient profiles be based on 100 g, 100 kcal or serving size? *Eur J Clin Nutr.* (2009) 63:898–904. 10.1038/ejcn.2008.53 18985061

