# Supplementary material for: Food-Level Analysis to Identify Dietary Choices With the Highest Nutritional Quality and Lowest Greenhouse Gas Emissions and Price
Source: Front Nutr. 2022 Apr 27;9:851826. doi: 10.3389/fnut.2022.851826 (PMC9094442; doi:10.3389/fnut.2022.851826)
Supplement: Supplementary file 1 [file Table_1.docx]

# Appendix.

**Supplemental Table 1.** Shapiro–Wilk test values among dimensions studied per 100 kcal

| **Category** | **NRF9.3** | **GHGE** | **Cost** |
| --- | --- | --- | --- |
| **All items** | W = 0.99524, p-value = 1.814e-11 | W = 0.54645, p-value < 2.2e-16 | W = 0.20872, p-value < 2.2e-16 |
| **Fruit and vegetables** | W = 0.9571, p-value = 1.555e-14 | W = 0.54036, p-value < 2.2e-16 | W = 0.61444, p-value < 2.2e-16 |
| **Potatoes, bread, rice, pasta and other starchy carbohydrates** | W = 0.9896, p-value = 3.286e-08 | W = 0.64448, p-value < 2.2e-16 | W = 0.35481, p-value < 2.2e-16 |
| **Beans, pulses, fish, eggs, meat and other proteins** | W = 0.9812, p-value = 4.539e-14 | W = 0.7607, p-value < 2.2e-16 | W = 0.28451, p-value < 2.2e-16 |
| **Dairy and alternatives** | W = 0.99099, p-value = 0.0227 | W = 0.68748, p-value < 2.2e-16 | W = 0.22126, p-value < 2.2e-16 |
| **Oils, spreads and fats** | W = 0.89381, p-value = 6.737e-06 | W = 0.90408, p-value = 1.818e-05 | W = 0.73947, p-value = 2.234e-10 |
| **Drinks** | W = 0.98589, p-value = 0.02606 | W = 0.5513, p-value < 2.2e-16 | W = 0.41501, p-value < 2.2e-16 |
| **Items that consumption should be limited** | W = 0.97388, p-value = 2.162e-05 | W = 0.70617, p-value < 2.2e-16 | W = 0.15579, p-value < 2.2e-16 |
